# Neo-adjuvant systemic radiation therapy for inoperable hepatic hilum neuroendocrine tumor with ^177^Lu- DOTATATE: successful final surgical resection

**DOI:** 10.22038/aojnmb.2025.85403.1614

**Published:** 2026

**Authors:** Ali Mohammad Moradi, Fatemeh Sharifian, Saeed Farzanefar, Mohammad Reza Ghasri, Mehrshad Abbasi

**Affiliations:** 1 Liver Transplantation Research Center, Imam Khomeini Hospital Complex, Tehran University of Medical Sciences, Tehran, Iran; 2 Department of Nuclear Medicine, Imam Khomeini Hospital Complex, Tehran University of Medical Sciences, Tehran, Iran

**Keywords:** Inoperable tumor treatment Radiation therapy, Neuroendocrine tumor resection, Neoadjuvant therapy A B S T R A C T

## Abstract

The role of neoadjuvant therapy in neuroendocrine tumors (NET) remains an area requiring further advanced clinical exploration. ^177^Lu-DOTATATE has demonstrated significant therapeutic efficacy in managing metastatic NET, with reports of notable tumor size reduction in specific cases. This report highlights the case of a young patient diagnosed with an initially inoperable hepatic hilum NET. The patient received two cycles of ^177^Lu-DOTATATE, resulting in remarkable tumor shrinkage on follow-up imaging, which facilitated surgical intervention. The patient underwent a complex left trisectionectomy resection, including anastomosis of the right portal vein, resection and interposition graft of the right hepatic artery, Roux-en-Y hepaticojejunostomy, and jejunojejunostomy. Due to residual involvement of the common hepatic duct and right portal vein margins, two additional cycles of adjuvant ^177^Lu-DOTATATE were administered post-surgery. Long-term follow-up imaging over a 20-month period has demonstrated stable disease, emphasizing the potential benefits of incorporating neoadjuvant and adjuvant ^177^Lu-DOTATATE in selected NET cases.

## Introduction

 Neoadjuvant chemoradiotherapy has demonstrated efficacy in selected tumors and specific clinical scenarios (1). It is particularly considered when tumor dimensions approach a threshold that may compromise operability, though surgical outcomes are anticipated to be suboptimal (2). Neoadjuvant treatments can facilitate tumor downstaging, potentially enabling surgical intervention (2, 3). Systemic radiation therapy using ^177^Lu-DOTATATE has been shown to be an effective treatment for metastatic neuroendocrine tumors, especially for gastroenteropancreatic (GEP) low-grade NETs (4, 5). Encouraging results have been observed in patients with progressive tumors unresponsive to Sandostatin. Emerging evidence suggests that ^177^Lu-DOTATATE may also be beneficial at earlier stages of disease when metastases remain responsive to Sandostatin. For patients with hepatic NET, tumor shrinkage following ^177^Lu-DOTATATE therapy has been documented (5, 6), suggesting potential effectiveness in primary hepatic NET cases. This case report details the treatment of a young patient with NET in the porta hepatis and biliary obstruction, initially deemed inoperable by a hepatobiliary and liver transplant multidisciplinary team, who subsequently underwent successful surgery following neoadjuvant ^177^Lu-DOTATATE therapy.

### Case Presentation

 A 28-year-old male presented in July 2022 with abdominal pain, jaundice, and pruritus. Initial lab results showed a total bilirubin level of 26 mg/dL (direct: 25 mg/dL). Abdominal computed tomography (CT) revealed a mass at the hepatic hilum, which further imaging by magnetic resonance imaging (MRI) and magnetic resonance cholangiopancreatography (MRCP) measured the tumor at 60×57 mm, with proximal common hepatic duct (CHD) invasion. Endoscopic retrograde cholangiopancreato-graphy (ERCP) was unsuccessful in stenting the CHD at the tumor site. Endoscopic ultrasound (EUS) showed proximal CHD wall thickening and mass formation; fine-needle aspiration (FNA) confirmed a neuroendocrine tumor (NET) with immunohistochemistry (IHC) findings positive for synaptophysin and chromogranin, with a Ki-67 index of 10% (Grade II). Percutaneous transhepatic stenting of the CHD reduced total bilirubin to 4.8 mg/dL (direct: 3.7 mg/dL), though jaundice and pruritus persisted.

**Figure 1:**
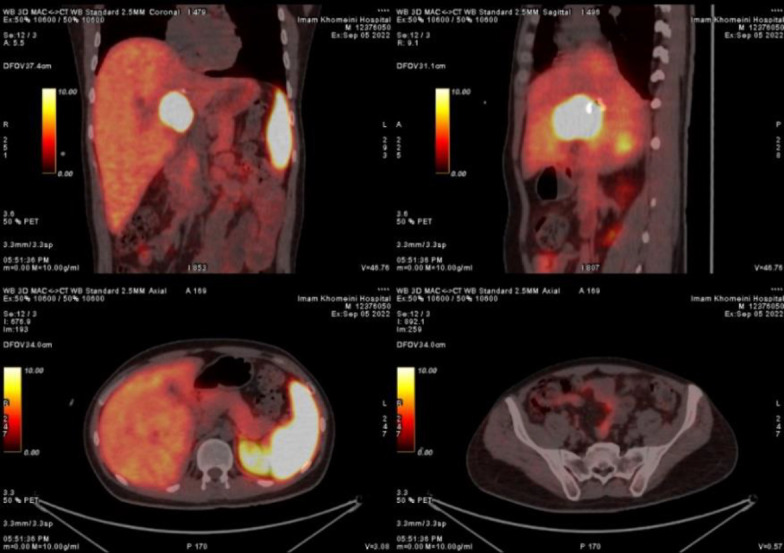
^68^Ga DOTATATE PET scan before the treatment indicating solitary DOTATATE avid liver hilum mass

 Upon referral to a university hospital, a multidisciplinary hepatobiliary and liver transplant team reviewed the case. MRI indicated extensive tumor involvement, including the portal vein, extending from the left to the right branches up to the second-order veins, as well as the hepatic artery up to its bifurcation, leading to an inoperable status. A nuclear physician suggested neoadjuvant ^177^Lu-DOTATATE therapy for potential tumor size reduction. PET/CT with ^68^Ga-DOTATATE showed a 60×47 mm avid mass at the hepatic hilum without extrahepatic involvement, and a small gallstone was noted (Figure 1). Laboratory findings included a white blood cell count of 6.4 k/dL, hemoglobin of 12.3 mg/dL, and platelets at 275 k/dL.

 The patient underwent two cycles of ^177^Lu -DOTATATE therapy (7.4 GBq per cycle) at 58-day intervals. Post-therapy imaging confirmed ^177^Lu -DOTATATE localization in the liver hilum tumor, with substantial tumor reduction to 28×30 mm after the second cycle. Clinical symptoms, including pain, jaundice, and pruritus, improved significantly following the initial cycle and resolved after the second. Total bilirubin levels dropped to 3.1 mg/dL (direct: 1.1 mg/dL) after the first cycle and further declined to 2.1 mg/dL (direct: 0.8 mg/dL) post-second cycle.

 Twelve days following the second ^177^Lu-DOTATATE treatment, contrast-enhanced CT (CECT) imaging showed an arterial-enhancing 25×42 mm mass involving the CHD, right bile duct, and right portal vein confluence, with sparing of the left portal vein and its branches. The hepatic artery showed tumor involvement extending into the right hepatic artery (25 mm). 

 The board recommended surgical intervention, including resection and anastomosis of the right hepatic artery, with preservation of the right portal vein.

 Surgery and Pathology Findings: A liver transplant surgeon performed a left trisectionectomy with extended caudate lobe resection, right portal vein resection and anastomosis, resection with interposition graft of the right hepatic artery, Roux-en-Y hepatico-jejunostomy, lymph node dissection, cholecystectomy, repair of the left hepatic vein, mesenteric repair, enteroclysis, jejuno-jejunostomy, and an omental flap placement.

 Post-surgical pathology confirmed a Grade I tumor at the common hepatic duct (CHD), measuring 24 mm, with extension into the bile duct and liver parenchyma. Perineural and lymphovascular invasion were observed, with positive margins at the CHD and right portal vein. All seven dissected lymph nodes were negative for malignancy. Additional findings included chronic cholecystitis, large bile duct obstruction, and ascending cholangitis in the liver tissue. Immunohistochemistry was positive for chromogranin and synaptophysin, with a Ki-67 index of less than 1%.

**Figure 2:**
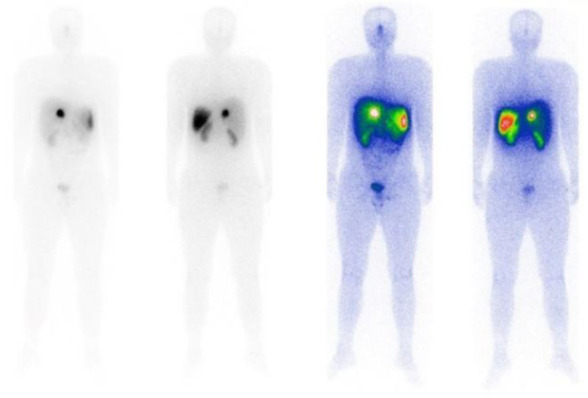
The whole-body scan after second cycle of ^177^Lu-DOTATATE therapy; the primary tumor localizes the radiopharmaceutical in hepatic hilum NET with significant shrinkage compared to the first cycle

**Figure 3:**
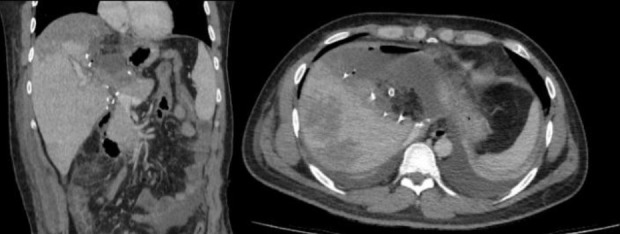
Post therapy CT scan indicating complete resection of the tumor with no evidence of remnant/metastatic disease

 Following surgery, two additional cycles of ^177^Lu -DOTATATE (7.4 GBq) were administered, with a 46-day interval before the first cycle and 65 days before the second. Post-therapy imaging was negative (Figure 2 and 3), and an Octreotide scan conducted in April 2023-10 months after the initial symptoms-showed no residual tumor at the liver hilum.

**Figure 4:**
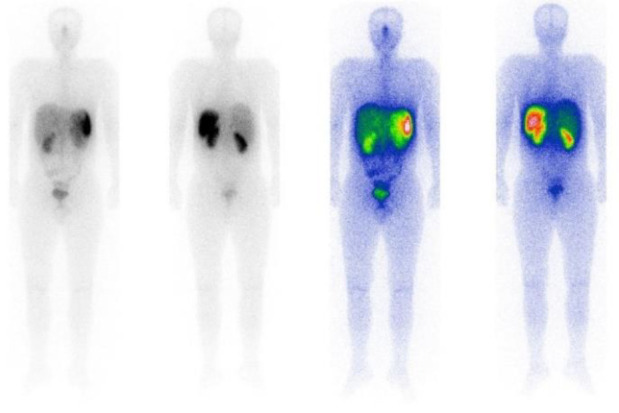
The whole-body scan after post-surgical ^177^Lu-DOTATATE therapy; no evidence of radiopharmaceutical localization is observed

 Follow-ups at 12 and 20 months indicated no recurrent or metastatic lesion. The patient was receiving sandostatin meanwhile (Figure 4).

## Discussion

 DOTATATE is a somatostatin analog that binds to somatostatin receptors (SSRs) on the cell surface (7). This molecule can be labeled with either imaging isotopes (e.g., ^99m^Tc or ^68^Ga) or therapeutic isotopes (e.g., ^177^Lu or^ 90^Y) (8). It is primarily approved for treating low-grade gastroenteropancreatic neuroendocrine tumors (GEP-NETs), but other neuroendocrine tumors, including medullary thyroid cancer, lung carcinoid, paraganglioma, and pheochromo-cytoma, express varying degrees of SSR and may benefit from DOTATATE-based radio-ligand therapy (RLT) (9, 10). Although low-grade tumors generally respond better to RLT, evidence suggests that high-grade NETs with SSR expression and DOTATATE uptake may also respond to ^177^Lu-DOTATATE therapy (11–13). The effectiveness of ^177^Lu-DOTATATE in metastatic cases suggests its potential for reducing tumor size preoperatively, a promising approach for NETs of borderline operability.

 In this case, we report a rare instance of symptomatic hepatic hilum NET rendered operable after two cycles of ^177^Lu-DOTATATE therapy. Initial symptoms disappeared, and tumor size reduced substantially, facilitating surgical resection. Successful operation was achieved despite minor residual involvement of the hepatic duct and right hepatic artery margins, which prompted adjuvant systemic radiation therapy. The final imaging demonstrated a complete response, under-scoring the potential of neoadjuvant RLT for downstaging certain malignancies. Although rare, similar application of radioligand therapy has been used in prostate cancer setting employing ^177^Lu-PSMA before prostatectomy. 

 Exceptionally, neoadjuvant ^177^Lu DOTATATE treatment in GEP-NETs have been documented; Parghane et al. (5) reported successful tumor shrinkage before operation of GEP-NET cases.

Two studies in the literature have evaluated the efficacy of ^177^Lu-DOTATATE therapy in patients with GEP-NETs, reporting improved resectability of previously inoperable pancreatic and gastrointestinal tumors. These studies included 31 and 57 patients, respectively, with inoperable GEPNETs-predominantly pancreatic in origin-with or without hepatic or intra-abdominal metastases (5, 14). The present case is unique due to the extraordinary location of the primary tumor at the hepatic hilum. A similar presentation in a pediatric patient was reported by Sougata et al (15).

 The use of ^177^Lu-DOTATATE in this instance supports the broader application of peptide receptor radionuclide therapy (PRRT), an alternative name for DOTATATE-based RLT, beyond its established role in GEP-NET management. Recent studies have reported favorable outcomes even in non-GEP-NETs with significant SSR expression (9, 10, 13). Our report suggests that PRRT could be a viable option for managing inoperable NETs in challenging anatomical sites, such as the hepatic hilum.

 One significant outcome of this therapy is its capability to provide symptomatic relief alongside tumor reduction. This patient experienced notable improvement in symptoms-including pruritus, jaundice, and abdominal pain-within one week of the initial ^177^Lu-DOTATATE cycle. Such rapid symptom relief is noteworthy, as it highlights RLT’s potential for improving quality of life even if operability remains limited. Supporting studies by Das et al. (4) and Mittra (7) have similarly reported substantial symptomatic improvement in NET patients treated with ^177^Lu-DOTATATE.

 Post-surgery, the patient received further ^177^Lu-DOTATATE cycles, with post-therapy imaging confirming the absence of active residual disease. The combination of neoadjuvant and adjuvant ^177^Lu-DOTATATE therapy may be transformative, particularly for cases with microscopic margin involvement, as seen here. This approach could reduce recurrence and micrometastasis risks, consistent with findings from previous PRRT neoadjuvant studies showing improved survival outcomes (4, 11, 12). Demirci et al. (13) demonstrated long-term survival benefits in patients receiving PRRT, even in those with high-grade NETs.

 In conclusion, this case supports the consideration of neoadjuvant ^177^Lu-DOTATATE in NETs with borderline operability and DOTATATE uptake. DOTATATE therapy offers the advantage of sequential imaging to monitor tumor response and metastatic spread after each cycle, aiding in surgical decision-making. 

 This case offers valuable insights into PRRT’s potential for treating NETs in challenging anatomical locations, such as the hepatic hilum, and highlights the need for further studies. PRRT’s unique imaging capabilities after each treatment cycle allow for dynamic monitoring, ensuring optimal timing and feasibility of surgical intervention.
